# Ultrasound super-resolution imaging for differential diagnosis of breast masses

**DOI:** 10.3389/fonc.2022.1049991

**Published:** 2022-11-03

**Authors:** Ge Zhang, Yu-Meng Lei, Nan Li, Jing Yu, Xian-Yang Jiang, Mei-Hui Yu, Hai-Man Hu, Shu-E Zeng, Xin-Wu Cui, Hua-Rong Ye

**Affiliations:** ^1^ Department of Medical Ultrasound, China Resources & Wisco General Hospital, Wuhan University of Science and Technology, Wuhan, China; ^2^ Hubei Province Key Laboratory of Occupational Hazard Identification and Control, Wuhan University of Science and Technology, Wuhan, China; ^3^ Department of Electrical and Electronic Engineering, Hubei University of Technology, Wuhan, China; ^4^ Department of Medical Ultrasound, Hubei Cancer Hospital, Tongji Medical College, Huazhong University of Science and Technology, Wuhan, China; ^5^ Department of Medical Ultrasound, Tongji Hospital, Tongji Medical College, Huazhong University of Science and Technology, Wuhan, China

**Keywords:** breast mass, super-resolution imaging, ultrasound, contrast-enhanced ultrasound, differential diagnosis

## Abstract

**Objective:**

Ultrasound imaging has been widely used in breast cancer screening. Recently, ultrasound super-resolution imaging (SRI) has shown the capability to break the diffraction limit to display microvasculature. However, the application of SRI on differential diagnosis of breast masses remains unknown. Therefore, this study aims to evaluate the feasibility and clinical value of SRI for visualizing microvasculature and differential diagnosis of breast masses.

**Methods:**

B mode, color-Doppler flow imaging (CDFI) and contrast-enhanced ultrasound (CEUS) images of 46 patients were collected respectively. SRI were generated by localizations of each possible contrast signals. Micro-vessel density (MVD) and microvascular flow rate (MFR) were calculated from SRI and time to peak (TTP), peak intensity (PI) and area under the curve (AUC) were obtained by quantitative analysis of CEUS images respectively. Pathological results were considered as the gold standard. Independent chi-square test and multivariate logistic regression analysis were performed using these parameters to examine the correlation.

**Results:**

The results showed that SRI technique could be successfully applied on breast masses and display microvasculature at a significantly higher resolution than the conventional CDFI and CEUS images. The results showed that the PI, AUC, MVD and MFR of malignant breast masses were significantly higher than those of benign breast masses, while TTP was significantly lower than that of benign breast masses. Among all five parameters, MVD showed the highest positive correlation with the malignancy of breast masses.

**Conclusions:**

SRI is able to successfully display the microvasculature of breast masses. Compared with CDFI and CEUS, SRI can provide additional morphological and functional information for breast masses. MVD has a great potential in assisting the differential diagnosis of breast masses as an important imaging marker.

## Introduction

Breast mass is one of the most common breast diseases globally. A number of studies have shown that more than 25% of women are affected by breast disease in their lifetimes (1). However, breast masses have a variety of causes, ranging from physiological adenosis to highly invasive malignancy. Breast cancer is the most frequently diagnosed cancer over the world. It is the leading cause of cancer death in women and poses a great public health challenge (2). Early diagnosis and treatment of breast cancer have an important impact on the prognosis of patients. Surgical excision and image-guided biopsy of breast masses are the gold standard for pathological evaluation of breast cancer. The commonly used methods include fine-needle aspiration biopsy, core-needle biopsy, and vacuum-assisted biopsy (3). But the invasiveness of biopsy and possible complications (such as bleeding, infection, etc.) make women uncomfortable.

It is known that angiogenesis appears to be important during cancer progression (4). Previous studies have shown that angiogenesis is a crucial factor affecting local invasion, growth and distant metastasis of breast cancer. The microvascular morphology and functional characteristics of benign and malignant breast masses are inconsistent (5). Related studies had shown that penetrating vessels, branching or disordered vessels may indicate malignant tumors (6). High micro-vessel density (MVD) in masses were more related to the possibility of invasive cancer metastasis and could significantly predict poor survival of breast cancer patients (7). More importantly, malignant breast masses usually have a higher MVD than benign masses (8). At present, the gold standard of MVD detection is obtained by vascular immunohistochemical staining, which is invasive for patients (9). Therefore, it is very important to explore a non-invasive imaging method to detect and evaluate microvasculature in breast masses.

Medical ultrasound imaging is regarded as one of the most common methods for breast masses imaging due to its noninvasiveness, accessibility, affordability (10). Ultrasound is of essential importance in breast cancer detection, diagnosis, and image-guided biopsy for a number of decades. Breast Imaging Reporting and Data System (BI-RADS) is widely used in breast cancer screening in a number of countries. It points out that breast tissue composition, calcification, shape, margin, orientation of masses are important factors to be considered in the classification of masses. However, it is impossible to detect the internal microvascular information of the masses only from the gray scale images. Ultrasound imaging techniques, such as color Doppler flow imaging (CDFI) and contrast-enhanced ultrasound (CEUS), can provide additional blood flow information for the diagnosis of breast masses.

CDFI is the common technique to detect the vascularity and flow information within breast masses noninvasively. It can detect the degree of vascularity within and around breast masses (11). Related studies (12, 13) have shown that most benign breast masses have less blood flow signals, the angiogenesis and the maximum flow velocity in malignant masses are higher than those in benign masses. However, it is challenging for CDFI to detect vessels with relatively slow flows (< 1 cm/s) and relatively small diameters (< 0.1mm) (14, 15). It is unable to display the true micro-vessels and microvascular flow rate (MFR) in the masses. Microvascular flow within the mass does not follow a constant and unidirectional path (16). Therefore, it requires a high-resolution imaging technique to observe the microvascular flow to monitor these functional changes within the masses.

CEUS has been widely used in clinical studies to visualize different vascular structures and tissues. The results showed that CEUS could provide more blood flow information than CDFI (17, 18). These parameters are helpful to differentiate benign and malignant masses and follow-up after local treatment. Some studies have shown that malignant masses tend to be rapid wash-in with hyper-enhancement, enlarged size, present penetrating vessels or crab claw-like pattern, while benign masses tend to be synchronous or slow wash-in with hypo-enhancement, with equal size after enhancement (19). However benign and malignant masses still have an overlap in contrast enhancement patterns. At present, there is no clear classification or guidance of CEUS to avoid the biopsy of indeterminate breast masses (20). Due to the acoustic diffraction limit of the operating ultrasound frequency and insufficient sensitivity, this technique is not capable to visualize the microvasculature at a micron scale. There is currently a lack of a non-invasive diagnostic imaging technique which can visualize microvascular features in clinical practice.

Over the last few years, inspired by optical super-resolution imaging (SRI), ultrasound SRI utilized the localizations of ultrasound contrast agents within blood vessels for the noninvasive display of microvasculature. The introduction of ultrasound SRI technique demonstrated the capability of breaking the ultrasound diffraction limit. After the injection of microbubble contrast agents, the individual microbubbles can be localized and tracked within a subwavelength resolution. The MVD and flow velocity within the masses can then be generated at a spatial scale of micrometers. Previous studies have demonstrated the application of this imaging technique in mouse ear, rat brain and various cancer model (21–23). Opacic et al. and Harput et al. had applied it on human for the first time (24, 25). Several studies had shown that ultrasound SRI technique could be successfully applied on human brain and kidney to further help clinicians for medical diagnosis (26–28). At the same time, ultrasound SRI had been proved to be able to visualize the complex microvasculature in human breast masses and provide rich information. Clinical feasibility of the method was demonstrated for chemotherapy monitoring of breast cancer, the ultrasound SRI can be used to access the early changes of breast cancer after treatment with a vascular-disrupting agent (29). Related researches obtained the MVD and super-resolved velocity map (SRVM) of the human breast masses through ultrasound SRI technique, which further verified the feasibility of using SRI to reveal the microvasculature in breast masses (15, 29). Therefore, by quantifying the extremely low flow rate of single vessels and displaying vascular characteristics at super-resolution, SRI is expected to significantly improve the differential diagnosis of breast masses and the monitoring of breast cancer response to treatment.

However, the feasibility of utilizing ultrasound SRI to differentiate human breast masses remains unknown. Therefore, this study aimed to explore the feasibility and the clinical value of ultrasound SRI compared with CDFI and CEUS images in the differential diagnosis of breast masses.

## Materials and methods

### Clinical data acquisition

**Figure 1:**
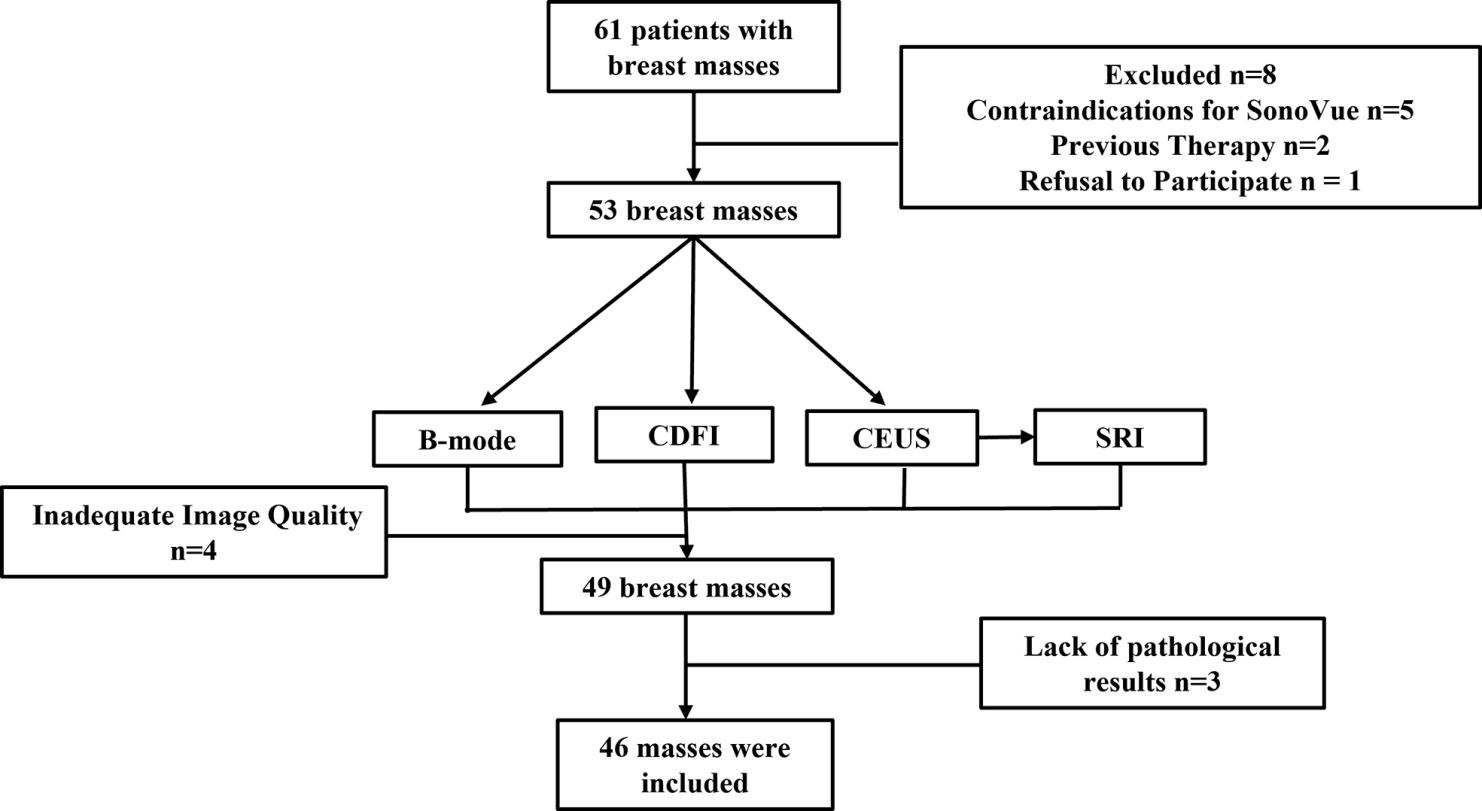
Flow chart of data acquisition.

The Institutional Review Board (IRB) of China Resources & Wisco General Hospital approved this study. Each patient was asked to sign an informed consent. The female patients over the age of 18 who were assessed as BI-RADS Category 4 or 5 with ultrasound were included in the study. This is because these patients had a greater clinical demand in CEUS examination for further diagnosis compared to the patients assessed as other BI-RADS Category. For patients with multiple suspicious masses, the most suspicious mass was chosen. Patients with contraindications for ultrasound contrast agents, previous therapy, psychiatric disorders or currently pregnant were excluded. All patients who meet the above requirements received B-mode, CDFI, CEUS examination. SonoVue (Bracco, Milan, Italy) microbubble contrast agent was selected as the contrast agent. Additionally, all patients underwent ultrasound-guided breast biopsy or surgical excision to obtain the histopathological results. In this preliminary study, a total of 46 breast masses of 46 women were enrolled between October 2021 and March 2022 (**Figure 1**).

All patients underwent breast ultrasound examination by the same radiologist with more than 15 years of experience. A commercial ultrasound system (Resona R9, Mindray Bio-Medical Electronics Co. Ltd., Shenzhen, China) and a L11-3U linear array probe (bandwidth: 3.0 MHz – 10.0 MHz) were used for both real-time ultrasound image monitoring and data acquisition. Patients were told to adopt supine position during the scanning, lift and abduct their arms to fully expose breasts and axilla. Additionally, the patients were asked to breathe calmly during the examination.

Breast was scanned under B-mode ultrasound, if any mass was found, the size, location, shape, orientation, margin, echogenicity, calcification, and posterior features of breast masses were observed and recorded. Meanwhile, CDFI was performed to observe the blood flow and vascular morphology in and around the masses. The B-mode and CDFI dataset were acquired respectively at the plane with the most abundant blood supply.

To acquire ultrasound SRI dataset, the CEUS imaging plane with the same region of interest as the B-mode was regarded as a reference imaging plane. Microbubble signals within the breast mass were monitored using dual-mode images after the injection of microbubble contrast agents. SonoVue microbubbles were administered intravenously as a bolus of 0.5 mL through a 19-gauge cannula in a peripheral vein. After the microbubble injection, the patients were asked to hold the breath for about 10 seconds. More than 1,000 CEUS images were collected at an average frame rate of 80 Hz. A mechanical index (MI) of 0.08 was used to avoid the microbubble destruction during the CEUS examinations. Then the acquired CEUS images were used for further ultrasound SRI processing to calculate MVD and MFR (See Section 2.2 for details).

For the routine CEUS examination, real-time dual-mode images (B-mode and CEUS) were used to guide the imaging plane and monitor the microbubble signals after the injection. The imaging plane was kept unchanged to make sure all the images were acquired within the same region. The routine clinical CEUS examination was conducted with the remaining 4.3 mL microbubble solution rapidly injected into the peripheral vein, 5 mL normal saline was injected immediately after it. And a timer and dynamic storage function were started while the contrast agent was injected. All the imaging parameters remain the same as the previous SRI examination except for the frame rate. Qualitative analysis of the contrast signals within the breast masses were performed to obtain various parameters, including time to peak (TTP), peak intensity (PI), area under the curve (AUC), etc. For each dataset, the quantitative parameters were compared with the pathological results.

### Ultrasound imaging processing

The DICOM image data exported from the ultrasound system was directly used for further SRI processing. The SRI program was performed off-line using MATLAB (MathWorks Inc., Natick, MA, USA). For each dataset, singular value decomposition (SVD) processing was applied on each image frame to further filter out the clutter and background signals. Super-Localization processing was performed on each image frame after SVD processing. Briefly, an image pixel value threshold was set to filter out the noise signals and thus obtain the microbubble signals. The values of area (A), intensity (I), and shape/eccentricity (E) of all the bubble signals were recorded. These indices were used to reject non-microbubble signals and noises. The coordinates of spatially isolated signals were obtained by the “centroid” method, which calculated the intensity-weighted center of the signals. All the localizations obtained from all the images were summed into the final SRI (25).

The MVD was defined as tracked microbubble area divided by the region of interest (ROI) area. The tracked microbubble area was defined as the total area of microvasculature displayed on SRI. The ROI was also manually drawn on MATLAB referring to the contours of benign and malignant breast masses on both the B-mode image and the corresponding SRI.

The super-resolved microvascular flow rate (MFR) was calculated based on the region of interest (ROI) which was manually drawn on MATLAB referring to the contours of benign and malignant breast masses on both the B-mode image and the corresponding super-resolved velocity map (SRVM). To compute the super-resolved MFR, the tracking method computes the best correlated bubble signals within the selected ROI between neighboring images. Briefly, each bubble signal detected in the frame K and each of the bubble signals in the frame K+1 were identified within a search window. Since the frame rated of 80 Hz used, 700 micrometers were set as the maximum search window so that flow rate up to 20 mm/s can be tracked. For each signal in the frame K, a matched signal in the frame K+1 was identified if they have the maximum normalized cross-correlation above an empirically determined threshold of 0.9.

### Statistical analysis

Statistical significance was computed to demonstrate the statistical differences of the corresponding parameters between benign and malignant group by t-test and chi-square test. A *P* value smaller than 0.05 was considered to indicate statistical significance; a *P* value smaller than 0.01, strong significance; and a *P* value small than 0.001, high significance. Independent chi-square test and multivariate logistic regression analysis were performed using the parameters. The chi-square test of independence was performed on the quantitative parameters PI, TTP, AUC of CEUS and the quantitative parameters MVD and MFR of super-resolution images respectively. The stepwise regression method was used to screen the independent variables. The variables were removed from the equation according to the results of Wald statistics. The inclusion criteria and exclusion criteria were both 0.10.

## Results

### Clinical information

Forty-six B-mode, CDFI and CEUS images from 46 patients with breast masses and pathological results were obtained. Ultrasound-guided core-needle biopsy was performed for 19 masses and surgical excision was performed for 27 masses. All 46 masses were pathologically evaluated with Hematoxylin-Eosin staining as the gold standard. The ultrasound characterizations of twenty benign masses and twenty-six malignant masses were shown in **Table 1** respectively. There was no significant difference in age, position, shape, orientation, and posterior features between benign and malignant masses (*P* > 0.05). The size, margin, echogenicity, and calcification of benign and malignant masses were statistically different (*P* < 0.05).

**Table 1:** Summary of clinical information from examined patients and the corresponding ultrasound characterizations of breast masses.

Findings	Benign	Malignant	t/χ^2^	*P*
**Number of masses**	20	26		
**Age**	43.75 ± 9.22	48.42 ± 9.27	-1.700	0.096
**Size(cm)**	1.72 ± 0.81	3.32 ± 1.45	4.740	0.000
**Position**			1.911	0.167
Right Left	11 (55.0) 9 (45.0)	9 (34.6) 17 (65.4)		
**shape**			2.448	0.118
Oval, round Irregular	6 (30.0) 14 (70.0)	3 (11.5) 23 (88.5)		
**Orientation**			3.782	0.052
Parallel, wider than tall Vertical, taller than wide	19 (95.0) 1 (5.0)	19 (73.1) 7 (26.9)		
**Margin**			11.944	0.001
Smooth Irregular, angular, spiculate	13 (65.0) 7 (35.0)	4 (15.4) 22 (84.6)		
**Echogenicity**			21.106	0.000
Hyperechoic, isoechoic Hypoechoic	12 (60.0) 8 (40.0)	0 (0.0) 26 (100.0)		
**Calcification**			16.138	0.000
Absent Microcalcification	18 (90) 2 (10)	8 (30.8) 18 (69.2)		
**Posterior features**			0.609	0.435
Enhancement, no changes Shadowing	19 (95.0) 1 (5.0)	23 (88.5) 3 (11.5)		

unless otherwise specified, data in parentheses are percentages.

### Ultrasound images of breast masses

CDFI、CEUS and SRI showed the blood vessels within the breast masses, while SRVM showed the microvascular flow rate within the masses. The B-mode images displayed the size, shape, margin, echogenicity and calcification of the breast masses. The CDFI images roughly showed the volume of blood flow and vascular morphology in and around the masses. The CEUS images revealed the perfusion homogeneity, enhancement range and the enhancement degree of masses compared to the surrounding tissue. The SRI and SRVM showed images the microvasculature and flow velocity within the breast masses respectively.

**Figure 2:**
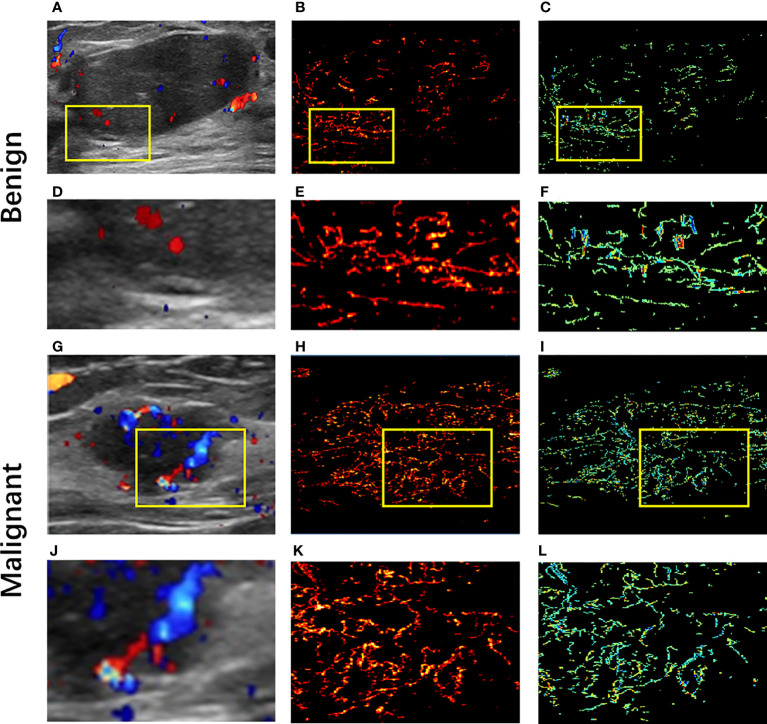
Ultrasound images of benign and malignant breast masses respectively. **(A, G)** Color-Doppler flow images of benign and malignant breast masses respectively; **(B, K)** super-resolution images of benign and malignant breast masses respectively; **(C, I)** super-resolved velocity images of benign and malignant breast masses respectively. **(D–F)** Zoomed-in sections as the yellow box indicated in **(A–C)**; **(J–L)** Zoomed-in sections as the yellow box indicated in **(G–I)**.

For CDFI, the results showed that the blood flow signals in malignant masses were more than that in benign masses, and the distribution of vascularization in malignant masses tended to be penetrating vessels while benign masses were peripheral vessels. **Figure 2** shows (A,G) CDFI images, (B,H) SRI images, the corresponding (C,I) SRVM images and (D–F,J–L) zoomed-in sections as the yellow box of benign and malignant breast masses respectively. CDFI can only show major vessels with relatively large diameters and fast blood flows since the limitation of poor signal-to-noise ratio and angle dependence as demonstrated in **Figures 2A, G**. CDFI was unable to detect blood flows with relatively low velocities due to the limitation of inability to distinguish motion artifacts from actual blood flow signals. However, SRI could offer detailed microvascular information that was unlikely to be visible in CDFI images. For example, Micro-vessels with diameters in micron-level could be observed in SRI, as shown in **Figures 2E, K**. Blood flow with much slower velocity could be detected in SRVM, as shown in **Figures 2F, L**. Similar to CDFI, the red and blue colors represented the relatively high flow rates in opposite directions and the yellow color represented relatively low flow rates in SRVM. Obviously, SRI can display more microvascular morphology and structure, and SRVM can show more information on microvascular flow velocity than CDFI.

**Figure 3:**
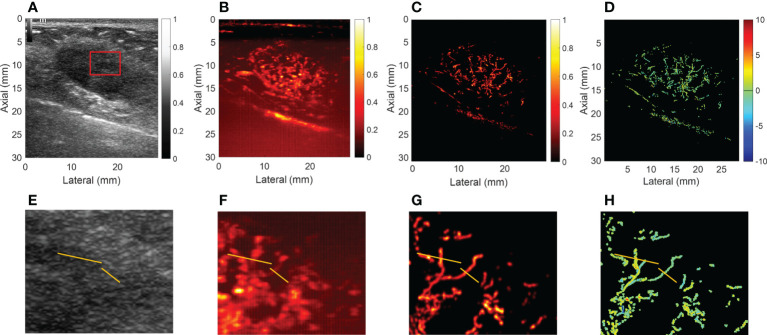
Ultrasound images of a representative benign breast mass. **(A)** B-mode image. **(B)** Contrast-enhanced ultrasound image. **(C)** Super-resolution image. **(D)** Super-resolved velocity image. **(E–H)** Shows the zoomed-in regions of **(A–D)** as the red box indicated in **(A)**. Yellow lines in **(E–H)** highlight the resolution improvement among the images.

**Figure 4:**
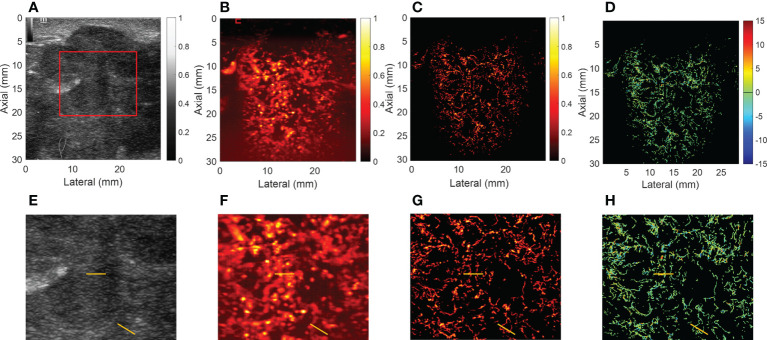
Ultrasound images of a representative malignant breast mass. **(A)** B-mode image. **(B)** Contrast-enhanced ultrasound image. **(C)** Super-resolution image. **(D)** Super-resolved velocity image. **(E–H)** shows the zoomed-in regions of **(A–D)** as the red box indicated in **(A)**. Yellow lines in **(E–H)** highlight the resolution improvement among the images.

For CEUS, the results showed that malignant breast mass had blurred edges and relatively high peak intensity compared with benign mass. **Figures 3**, **4** showed the (A) B-mode and the corresponding (B) CEUS, (C) SRI, and (D) SRVM images of a representative benign and malignant breast mass. **Figures 3E–H** showed the zoomed-in regions of **Figures 3A–D** as the red box indicated in **Figure 3A**. The same is true of **Figure 4**. However, due to the spatial resolution limitation of the conventional ultrasound, it is unlikely to clearly display the microvascular architecture in tumors as demonstrated in **Figures 3B, F** and **Figures 4B, F**. SRI was capable to break the ultrasound diffraction limit and significantly improve spatial resolution. After super-localization processing, as demonstrated in **Figures 3G, H** and **Figures 4G, H**, two adjacent micro-vessels and the tortuosity of micro-vessels in mass were clearly visualized in SRI whereas they cannot be observed on CEUS. SRI can reveal the microvasculature in breast masses with a much greater detail than CEUS.

### Comparison of quantitative parameters

**Figure 5:**
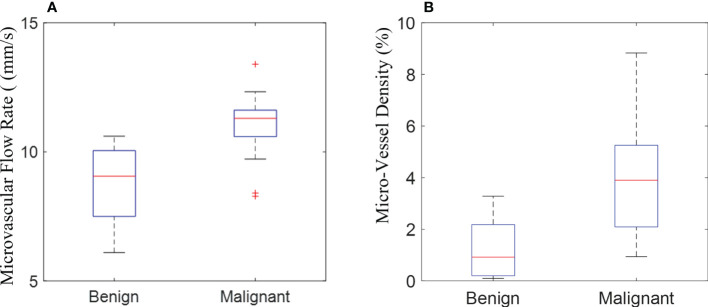
The box plot shows the quantification of microvascular flow rate **(A)** and micro-vessel density **(B)** measured in the super-resolution imaging between benign and malignant breast masses.

The quantitative parameters, MVD and MFR were computed from the SRI. The other quantitative parameters (PI, TTP, and AUC) were computed from CEUS images. In addition, the correlations between all the quantitative parameters of breast masses and the corresponding pathological results were further compared. The inspection results obtained were shown in **Table 2**. The box plots of MVD and MFR in both benign and malignant breast masses can be seen in **Figure 5**. It was found that the average PI of benign and malignant breast masses were 7.023 ± 4.199 dB and 15.181 ± 5.953 dB (*p* < 0.001) respectively. The average TTP were 29.178 ± 15.388 s and 23.238 ± 7.348 s (*p* < 0.001) respectively, and the average AUC were 168.399 ± 179.158 dB/s and 537.039 ± 449.546 dB/s (*p* < 0.001) respectively. The mean MFR of benign and malignant masses were 9.057 ± 1.696 mm/s and 10.381 ± 1.527 mm/s respectively (*p* < 0.001). The mean value of the MVD in benign breast masses was 0.948 ± 0.991% whereas that in malignant breast masses was 3.668 ± 2.019% (*p* < 0.001). Both differences between benign and malignant breast masses were statistically significant. The results showed that the PI, AUC, MVD and MFR of malignant breast masses were significantly higher than those of benign breast masses, while TTP of malignant breast masses was significantly lower than that of benign breast masses.

**Table 2:** Independent chi-square test of quantitative parameters in the diagnosis of benign and malignant breast masses.

Parameters	Mean (Benign)	Std. (Benign)	Mean (Malignant)	Std. (Malignant)	Chi-Square	*P*
**PI**	7.023	4.199	15.181	5.953	87.505	<0.001
**TTP**	29.178	15.388	23.238	7.348	123.974	<0.001
**AUC**	168.399	179.158	537.039	449.546	154.338	<0.001
**MFR**	9.057	1.696	10.381	1.527	56.872	<0.001
**MVD**	0.948	0.991	3.668	2.019	98.545	<0.001

Multivariate logistic regression analysis of quantitative parameters in the differential diagnosis of breast masses was performed. As shown in **Table 3**, the results showed that only PI and MVD finally entered the regression equation. The odds ratio (OR) was an index reflecting the correlation strength. The results showed that he OR for PI was 1.520 (*p* = 0.018, 95% C.I. = 1.074 ~ 2.149), and the OR for MVD was 8.525 (*p* = 0.042, 95% C.I. = 1.076 ~ 67.563). The OR value of MVD was higher than that of PI, which means that the MVD had a more significant correlation in differentiating the benign and malignant breast masses. This result suggested that SRI could display more detailed microvascular features between benign and malignant breast masses than CEUS

**Table 3:** Multivariate logistic regression analysis of quantitative parameters in the diagnosis of benign and malignant breast masses.

Parameters	β	S.E.	Wald	*P*	OR	95% C.I. for OR	
						Lower	Upper
**PI**	0.419	0.177	5.599	0.018	1.520	1.074	2.149
**MVD**	2.143	1.056	4.117	0.042	8.525	1.076	67.563
**Constant**	-8.262	3.571	5.354	0.021	0.001	–	–

## Discussion

Ultrasound SRI is a new emerging technique inspired from optical SRI (30, 31). A number of previous studies had demonstrated the capability of applying ultrasound SRI technique in various animals and in human (21–23, 25, 32–34). The value of SRI in differential diagnosis of breast masses remains to be studied. This study applied ultrasound SRI technique on humans and explored its clinical feasibility and diagnostic value in the differential diagnosis of breast masses compared with CEUS and CDFI images. B-mode, CDFI, CEUS and SRI examinations were performed on 46 breast masses respectively. Quantitative parameters were extracted from different datasets and compared with pathological results. The results demonstrated the feasibility of using SRI to visualize microvasculature in human breast masses. Additionally, SRI could display micro-vessels of breast masses at a significantly higher resolution than the conventional CDFI and CEUS images. Furthermore, the MVD and MFR, as quantitative parameters for evaluating the microvasculature, were successfully quantified from high quality SRI and SRVM images respectively. Compared with quantitative parameters (PI, TTP, AUC) obtained from CEUS, MVD has higher diagnostic value in differentiating benign and malignant breast masses.

The feasibility of SRI to display the microvasculature in breast masses at higher resolution relative to CEUS and CDFI was further validated. The result is consistent with the previous studies (15). Relevant studies showed that CDFI could be used to monitor blood flow and perfusion in tissues, but it only provided macro-blood flow information. The actual low-speed blood flow and movement artifacts cannot be distinguished. Therefore, the ability of CDFI to evaluate micro-vessels and microvascular flow rate was very limited (35, 36). CEUS visualizes microcirculation that is usually invisible in CDFI by detecting the enhanced backscatter echo of microbubbles (37). However, due to insufficient sensitivity and the acoustic diffraction limit of the operating US frequencies, neither technique provides a high enough spatial resolution to assess micro-vessels. Ultrasound SRI could achieve a high spatial resolution of vasculature beyond the acoustic diffraction limit (38). Dencks et al. (29) studied the feasibility of SRI for microvascular detection of clinical breast masses in 2019. The results demonstrated that microbubbles tracking-based ultrasound SRI provided visualizations and information beyond the CEUS technique. Ghost et al. (39) and Opacic et al. (24) also proved that ultrasound SRI technology can visualize and characterize the changes of microvascular network in the treatment response of breast cancer mice and patients respectively. These studies fully demonstrated the ability of SRI to show the microvascular of breast masses.

Ultrasound SRI can not only display the micro-vessels of breast masses at the capillary level, but also obtain more information through the quantitative analysis, such as MVD and MFR. In this study, MVD and MFR in breast masses were calculated based on the generation of ultrasound SRI and SRVM images. Chen et al. (38) compared the SRI with histology in MVD estimation of sham kidneys, contralateral kidneys, and injured kidneys at 21-days post injury and injured kidneys at 42-days post injury respectively. The results showed that there was a significant correlation between MVD measured in histology and SRI in the same area, which supported the accuracy of SRI in evaluating micro-vessel density. Song and his colleagues also demonstrated the microvascular perfusion image and blood flow velocity image *in vivo* rabbit kidney model through SRI technology to calculate MVD and MFR (40). Huang et al. used SRI technology to display the microvascular velocity map in the chorioallantoic membrane of chicken embryos and obtained the MFR (41). Previous studies have proved the accuracy of calculating MVD and MFR using ultrasound SRI.

In addition, quantitative analysis on CEUS and ultrasound SRI were performed to evaluate the ability of each parameter to differentiate between malignant and benign breast masses. It was found that all five parameters (PI, TTP, AUC, MVD and MFR) had statistical differences, among which MVD had the highest correlation. For CEUS, the results showed that the PI and AUC of malignant breast masses were significantly higher than those of benign breast masses, while TTP of malignant breast masses was significantly lower than that of benign breast masses. These findings can be explained by the earlier, faster and higher enhancement of malignant masses. This result showed the consistency with a number of previous studies (3). The research of Jung et al. indicated the CEUS perfusion parameters PI and AUC provided more information for assessing the risk of malignant breast masses (42). Janu et al. also proved that malignant masses showed statistically significantly lower TTP parameters than benign masses (18). For SRI, the results suggested that the MVD and MFR within the malignant breast masses were significantly higher than those within the benign breast masses. This may be related to the high density of blood vessels and the high incidence of arteriovenous shunts in breast malignant masses. It is well-known that angiogenesis is a hallmark of cancer and promotes tumor progression and metastasis (43). Relevant studies have shown that MVD is often regarded as a surrogate marker of angiogenesis in tumors (44) and is considered as a risk factor of metastasis and predicts poor prognosis of breast cancer patients (45). The Felix et al. determined MVD by counting the CD31-positive vessels in sections of breast biopsies and found that women with *in situ* or invasive breast cancer were more likely to have higher tissue MVD than women with benign masses. MVD, as a risk factor of breast cancer, is positively associated with breast cancer incidence (46). Studies of Krüger et al. (43) and Tolaney et al. (47) implicated that MVD could significantly predicted response to neoadjuvant in breast cancer. In addition, SRI of the tumor vasculature had also been clinically verified for chemotherapy monitoring of breast cancer (24, 29). However, there is no study to distinguish malignant masses from benign masses by showing the microvascular networks in breast masses using SRI. This study showed that SRI had a potential to become a new imaging method for differential diagnosis of breast masses by revealing microvascular and calculating quantitative indicators (such as MVD and MFR).

There are some limitations existing in this study. First, a larger sample size may be required to obtain a more solid conclusion to differentiate the benign and malignant breast masses using ultrasound SRI. However, the data acquisition of SRI dataset is challenging as this applies to all SRI studies on humans (15, 29). There were only less than 10 SRI cases shown in the previous studies. Second, the SRI generation is time-consuming and requires off-line processing. The graphics processing unit (GPU) parallel processing may be required to be integrated into the SRI processing in the future to further accelerate the SRI processing. Third, the ultrasound SRI and SRVM obtained in this study were two-dimensional imaging, therefore, the out of plane microvasculature could not been revealed. A 3D SRI technique equipped with a 2D array probe is expected to overcome this problem in the future. Finally, pathological underestimation may happen using ultrasound-guided breast biopsy. This is because either ultrasound-guided breast biopsy or surgical excision was used in this study to obtain the histopathological results.

In conclusion, it is feasible for SRI to show the details of micro-vessels in breast masses at a submicron resolution level compared with CDFI and CEUS. Meanwhile, more micro-vessel information can be obtained by calculating MVD and MFR. The MFR and MVD within the malignant breast masses were significantly higher than those within the benign breast masses respectively. Compared with the quantitative parameters of CEUS, MVD had a stronger significance in differentiating benign and malignant breast masses. This work provides an improved approach for the differential diagnosis of breast masses besides conventional B-mode, CDFI and CEUS images. With the study of breast masses with larger sample size, and the extraction of more microvascular parameters, ultrasound SRI technology is expected to further contribute to the classification of different pathological types of breast masses in clinics.

## Data availability statement

The raw data supporting the conclusions of this article will be made available by the authors, without undue reservation.

## Ethics statement

The studies involving human participants were reviewed and approved by The Institutional Review Board (IRB) of China Resources & Wisco General Hospital. The patients/participants provided their written informed consent to participate in this study. Written informed consent was obtained from the individual(s) for the publication of any potentially identifiable images or data included in this article.

## Author contributions

GZ, Y-ML, NL, JY, X-YJ, M-HY, and H-MH contributed to the collection of relevant literature. Y-ML and NL contributed to the literature analysis and manuscript preparation. GZ and Y-ML sorted out the literature and wrote the manuscript. S-EZ, X-WC, and H-RY were responsible for design of this article and provided data acquisition, analysis, and interpretation. All authors contributed to the article and approved the submitted version.

## Funding

This work was supported by the Key Research and Development Project of Hubei Province (No.2020BCB022), the joint fund project of the Hubei Provincial Health and Family Planning Commission (No. WJ2019H197) and the research project of Wuhan Municipal Health Commission (No.WX21C07).

## Acknowledgments

The authors would like to thank the helps from Prof. Mengxing Tang at Imperial College London in the completion of this article and the reviewers for reviewing this article.

## Conflict of interest

The authors declare that the research was conducted in the absence of any commercial or financial relationships that could be construed as a potential conflict of interest.

## Publisher’s note

All claims expressed in this article are solely those of the authors and do not necessarily represent those of their affiliated organizations, or those of the publisher, the editors and the reviewers. Any product that may be evaluated in this article, or claim that may be made by its manufacturer, is not guaranteed or endorsed by the publisher.
